# The effect of surgeon-controlled variables on construct stiffness in lateral locked plating of distal femoral fractures

**DOI:** 10.1186/s12891-021-04341-2

**Published:** 2021-06-04

**Authors:** Michael J. Weaver, George W. Chaus, Aidin Masoudi, Kaveh Momenzadeh, Amin Mohamadi, Edward K Rodriguez, Mark S. Vrahas, Ara Nazarian

**Affiliations:** 1 Department of Orthopaedic surgery, Brigham and Womens Hospital, 75 Francis Street, MA 02115 Boston, USA; 2 Frontrange Orthoaedics and Spine, 1610 Dry Creek Drive, CO 80503 Longmont, USA; 3 Musculoskeletal Translational Innovation Initiative, Carl J. Shapiro Department of Orthopaedic Surgery, Beth Isreal Deconess Medical Center, 330 Brookline Ave, MA 02215 Boston, USA; 4 Cedars-Sinai Medical Center, 8700 Beverly Blvd, CA 90048 Los Angeles, USA; 5 Department of Orthopaedic Surgery, Yerevan State Medical University, Yerevan, Armenia

**Keywords:** Supracondylar Femur, Distal Femur, Nonunion, Biomechanics, Lateral Locked Plating, Fracture, LISS

## Abstract

**Background:**

Nonunion following treatment of supracondylar femur fractures with lateral locked plates (LLP) has been reported to be as high as 21 %. Implant related and surgeon-controlled variables have been postulated to contribute to nonunion by modulating fracture-fixation construct stiffness. The purpose of this study is to evaluate the effect of surgeon-controlled factors on stiffness when treating supracondylar femur fractures with LLPs:

1. Does plate length affect construct stiffness given the same plate material, fracture working length and type of screws?
2. Does screw type (bicortical locking versus bicortical nonlocking or unicortical locking) and number of screws affect construct stiffness given the same material, fracture working length, and plate length?
3. Does fracture working length affect construct stiffness given the same plate material, length and type of screws?
4. Does plate material (titanium versus stainless steel) affect construct stiffness given the same fracture working length, plate length, type and number of screws?

**Methods:**

Mechanical study of simulated supracondylar femur fractures treated with LLPs of varying lengths, screw types, fractureworking lenghts, and plate/screw material. Overall construct stiffness was evaluated using an Instron hydraulic testing apparatus.

**Results:**

Stiffness was 15 % higher comparing 13-hole to the 5-hole plates (995 N/mm849N vs. /mm, *p* = 0.003). The use of bicortical nonlocking screws decreased overall construct stiffness by 18 % compared to bicortical locking screws (808 N/mm vs. 995 N/mm, *p* = 0.0001). The type of screw (unicortical locking vs. bicortical locking) and the number of screws in the diaphysis (3 vs. 10) did not appear to significantly influence construct stiffness (*p* = 0.76, *p* = 0.24). Similarly, fracture working length (5.4 cm vs. 9.4 cm, *p* = 0.24), and implant type (titanium vs. stainless steel, *p* = 0.12) did also not appear to effect stiffness.

**Discussion:**

Using shorter plates and using bicortical nonlocking screws (vs. bicortical locking screws) reduced overall construct stiffness. Using more screws, using unicortical locking screws, increasing fracture working length and varying plate material (titanium vs. stainless steel) does not appear to significantly alter construct stiffness. Surgeons can adjust plate length and screw types to affect overall fracture-fixation construct stiffness; however, the optimal stiffness to promote healing remains unknown.

## Background

Distal femur fractures are a common orthopaedic problem with an overall incidence of approximately 37 per 100,000 person-years [1]. These injuries occur in younger patients with high-energy trauma, and increasingly in older patients after low-energy mechanisms. Further, the number of these fractures are increasing around total knee replacements, as the rate of total knee arthroplasty has increased [2]. With the advent of anatomically contoured, locked plates, lateral locked plating (LLP) of the femur has become a common treatment of these injuries [3]. Initial studies of LLP for supracondylar femur fractures have been previously reported with very low rates of nonunion (0–14 %) [4–17]. However, more recent studies have indicated that nonunion rates may be much higher (up to 17–21 %) [18–20]. These fractures are often associated with decreased callus formation and problems with healing in up to 32 % of the patients [21]. Patients and injury related factors such as obesity, diabetes, smoking and open fracture have been associated with increased rates of nonunion [22].

Various implant related factors have been postulated to contribute to nonunion, presumably through the effect of modulating the overall construct stiffness. Among these, implant material (stainless steel vs. titanium), plate length, screw type, and screw density have received particular scrutiny [23, 24]. There is an emerging body of literature suggesting that construct patterns that are too stiff may contribute to increased risk of nonunion [20, 21, 24]. While many agree that stiffness is likely an important factor in creating a biomechanical environment conducive to fracture healing, the ideal stiffness has yet to be identified. Further, the effect of surgeon controlled factors, such as plate length, screw type and number, fracture working length and plate material, on the overall fracture-fixation construct stiffness have not been thoroughly evaluated.

The purpose of this study is to evaluate the effect of surgeon-controlled factors on overall fracture-fixation construct stiffness when applying lateral locked plates in the treatment of supracondylar femur fractures:

1. Does plate length affect construct stiffness given the same plate material, fracture working length and type of screws?
2. Does screw type (bicortical locking versus bicortical nonlocking or unicortical locking) and number of screws affect construct stiffness given the same material, fracture working length, and plate length?
3. Does fracture working length affect construct stiffness given the same plate material, length and type of screws?
4. Does plate material (titanium versus stainless steel) affect construct stiffness given the same fracture working length, plate length, type and number of screws?

## Methods

We performed a mechanical study on simulated supracondylar femur fractures using biomechanical femur models to evaluate the effect of surgeon-controlled variables on implant stiffness in 6 conditions.

**Fig. 1:**
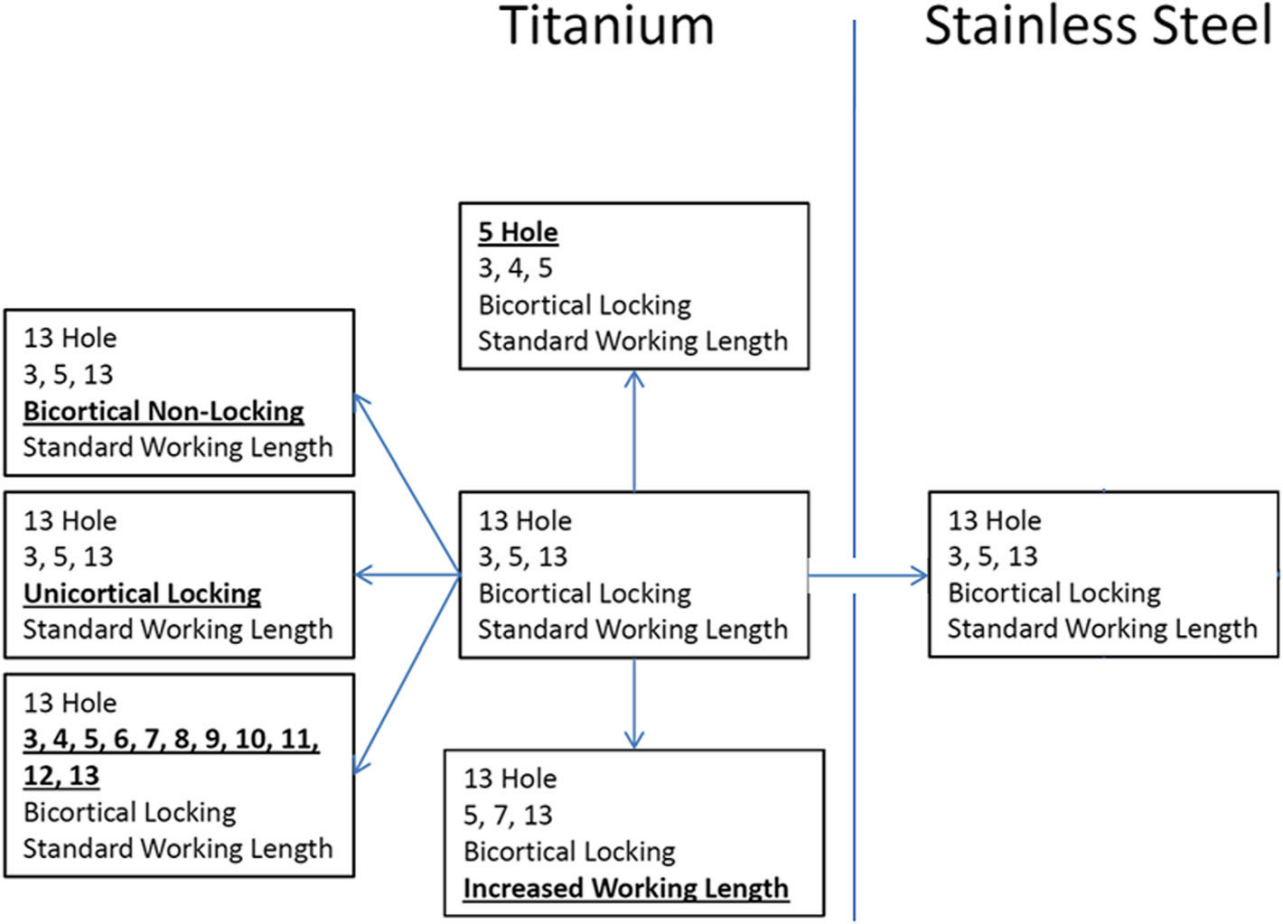
A diagram representing the different fracture-fixation constructs tested with the comparisons made. In each case the bold/underlined variable is the change made from the standard testing model. All constructs were tested in both titanium and stainless steel to compare the effect of plate and screw material

**Fig. 2:**
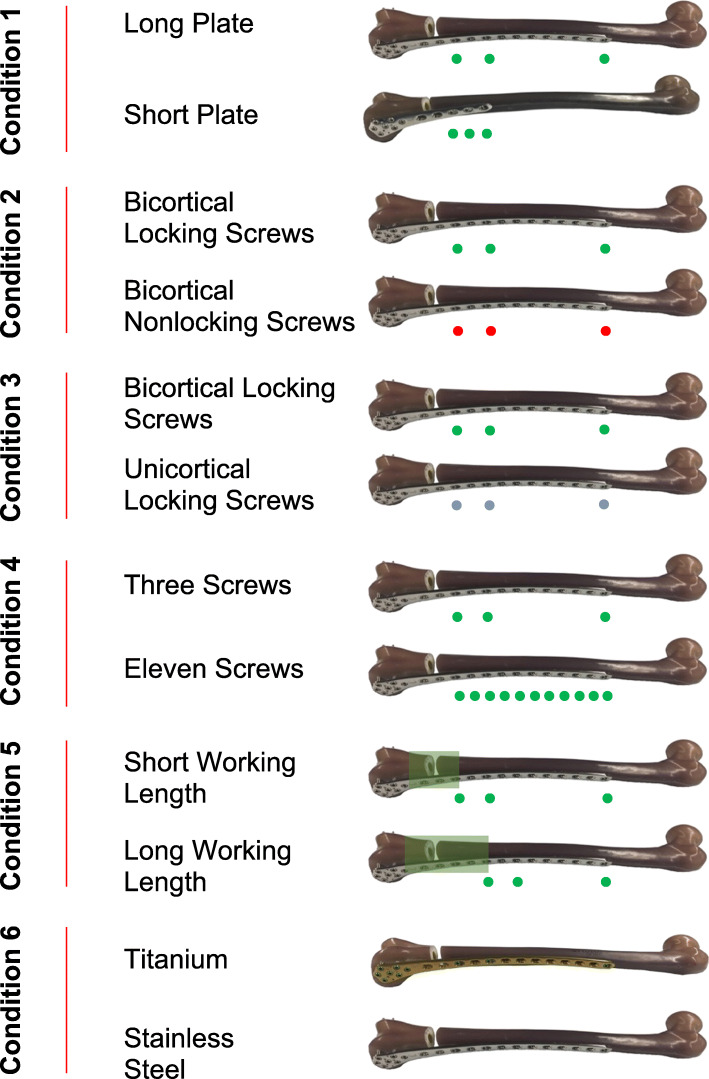
An illustration of the various constructs tested indicating plate length, screw type and pattern, fracture working length and plate material

In order to examine the effect of plate length, screw types, number of screws, fracture working length and plate material 7 different configurations were tested (Fig. 1). The “standard” construct was a 13-hole titanium distal femur lateral locked plate, Synthes Less Invasive Stabilization System (LISS) (Paoli, Pennsylvania, USA) plate, with 5mm diameter bicortical locking screws filling holes 3, 5, and 13. All 7 holes in the distal metaphyseal cluster were filled for all constructs using 5mm diameter bicortical locking screws. Other constructs were created to vary a single variable compared to the “standard” construct (Fig. 2). To test the influence of plate length on construct stiffness, a 5-hole plate was used with screws in holes 3, 4 and 5 (Condition 1). To test the effect of screw type and number the proximal bicortical locking screws were compared to constructs with bicortical nonlocking screws, unicortical locking screws, adding and filling all the holes of the plate with bicortical locking screws (Conditions 2, 3 and 4). Fracture working length was compared by increasing the distance from the fracture to the first hole filled with a screw by using the 5, 7, and 13 holes (Condition 5). Each of these previous configurations was repeated with stainless steel plates and screws to examine the effect of material on stiffness (Condition 6).

Large size 4th Generation Biomechanical Sawbone Femur Models were used (Vashon Island, Washington, USA), as our goal was to assess relative changes in construct stiffness in a consistent manner. These composite analougues femurs simulate structural properties and geometries of human bone, with a unique short fiber epoxy shell that mimic cortical bone which is filled with polyurethane foam resembling the bone marrow and cancellous bone at the epiphysis [25–27]. All bones were instrumented using titanium alloy Less Invasive stabilization system (LISS) plates or stainless steel condylar locking plates (Synthes, West Chester, PA). Plates were positioned in the same position for each construct. The distal end of the plate positioned against the lateral condyle while the proximal end remained in contact with the bone shaft. All plates were compressed to the bone by external clamps prior to screw insertion and all screws were tightened similarly using a 4.0 Nm torque wrench to ensure a uniform degree of tightening. After plate fixation, a one-centimeter gap between the first and the second holes proximal to the condylar part of the plate,was cut parallel to the distal femoral condyle axis to mimic a comminuted extra-articular supracondylar femoral fracture (OTA/AO type 33-A3) [28].

**Fig. 3:**
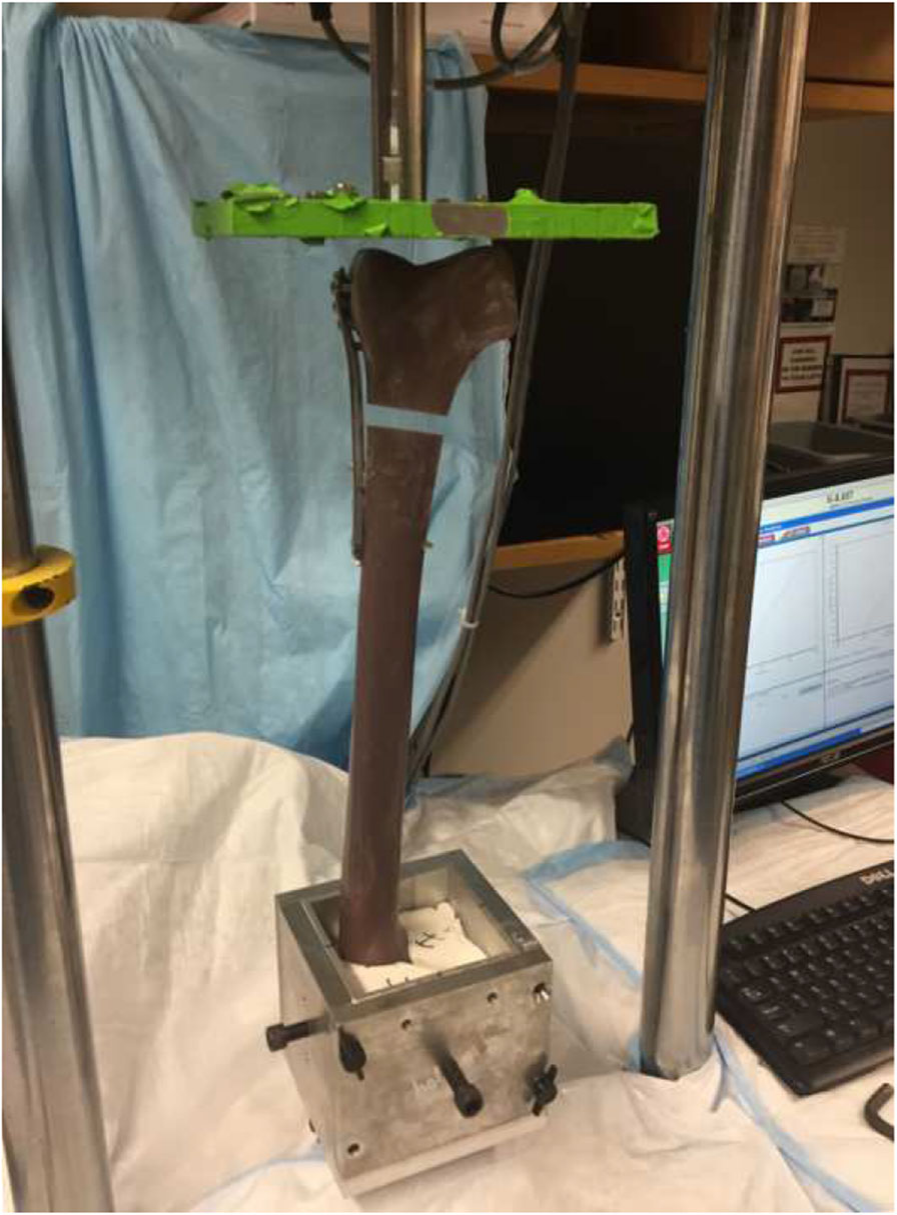
The mechanical testing apparatus used for the study

The distal femur constructs were tested for overall construct stiffness (K, N/mm) in the axial direction. The proximal ends of the femurs were potted in a polyurethane elastomer and were placed in a custom-made jig with a shear relief apparatus in the vertical position. Axial compression from the distal end was applied to the constructs on an Instron hydraulic testing apparatus (Instron 8511, Norwood, MA, USA) (Fig. 3). Each construct was tested with five specimens, with each specimen being tested three times. A displacement-controlled test with a rate of 1 mm per second was applied to the constructs with an end point of load 10 % lower than the yield load. Stiffness was calculated as the slope of the load-displacement curve between 20 and 60 % of the yield load (in the linear region).

Prior to the initiation of the study, we tested 5 intact and then instrumented models to determine stiffness values and to calibrate the testing jig. Based on this pilot study, five specimens per group were considered adequate to detect a 20 % change in the stiffness with 80 % power (nQuery Advisor, version 7.0, Statistical Solutions, Saugus, MA, USA). A repeated measure analysis of variance (ANOVA) with stiffness as the dependent variable, group as fixed factor, while accounting for multiple measures from the same specimen (each test conducted in triplicate), was conducted using SPSS v23 (IBM SPSS, Armonk, NY, USA). Two-tailed values of *p* < 0.05 were considered statistically significant.

## Results

**Fig. 4:**
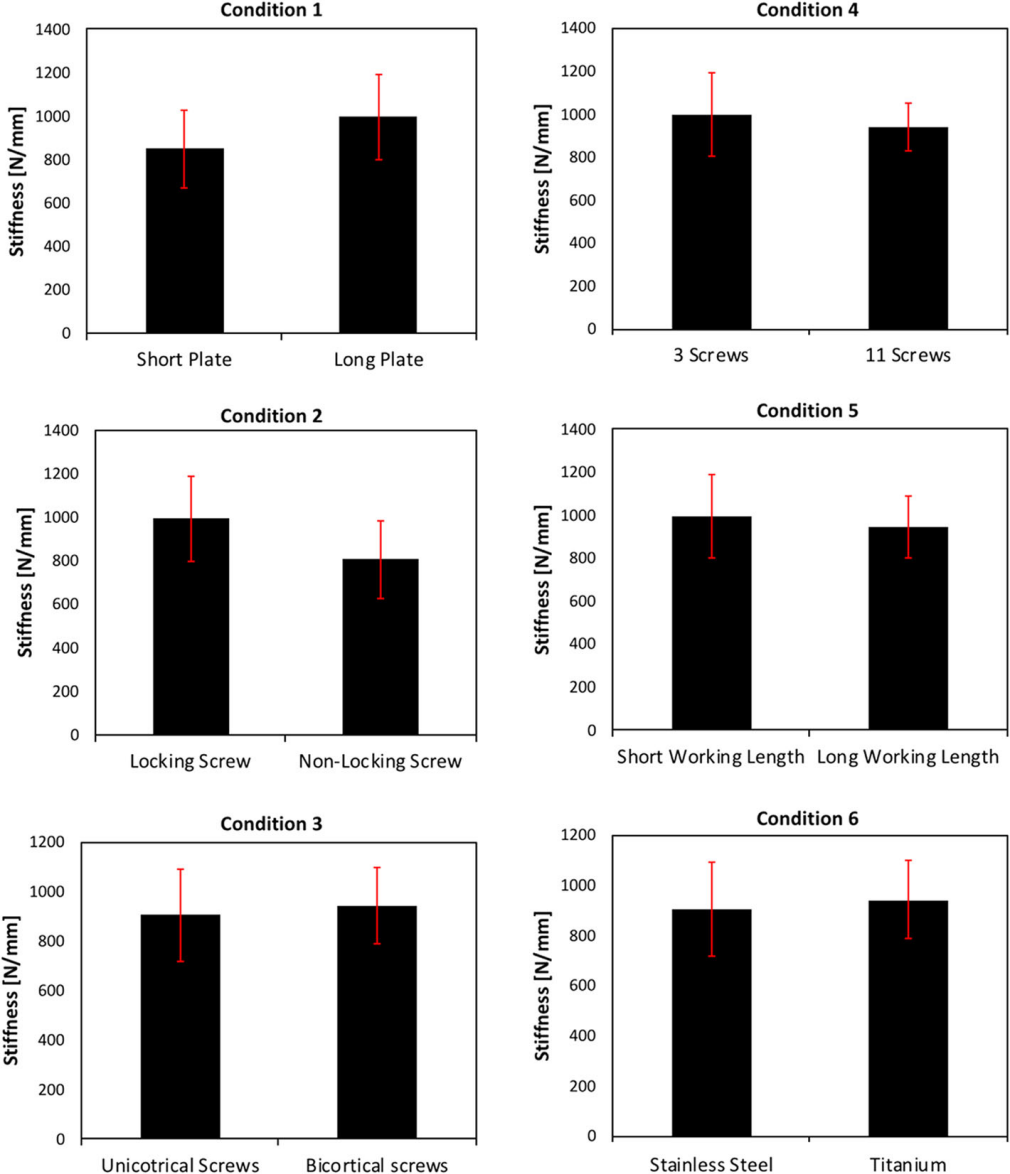
Comparisons of overall fracture-fixation construct stiffness. **a** Condition 1: 5-hole plate compared to 13-hole plate. **b** Condition 2: bicortical locking screws compared to bicortical nonlocking screws. **c** Condition 3: bicortical locking screws compared to unicortical locking screws. **d** Condition 4: 3 screws compared to 11 screws filling the proximal portion of the plate. **e** Condition 5: short fracture working length compared in longer fracture working length. **f** Condition 6: stainless steel plate and screw constructs compared to titanium plate and screw constructs

Does plate length affect construct stiffness given the same plate material, fracture working length and type of screws? Yes, longer plate length appears to have a significant influence on stiffness when other surgeon-controlled variables are kept constant. Stiffness was 15 % higher comparing the 13-hole and the 5-hole plates (995 N/mm849N vs. /mm, *p* = 0.003) (Fig. 4a). Longer plates generate a stiffer overall fracture-fixation construct.

Does screw type (bicortical locking versus bicortical nonlocking or unicortical locking) and number of screws affect construct stiffness given the same material, fracture working length, and plate length? The use of bicortical nonlocking screws decreased overall construct stiffness by 18 % compared to bicortical locking screws (808 N/mm vs. 995 N/mm, *p* = 0.0001) (Fig. 4b). The use of unicortical as opposed to bicortical locking screws did not appear to influence overall stiffness. The stiffness of the constructs with unicortical locking screws (995 N/mm) was similar to those with bicortical locking screws (1006 N/mm, *p* = 0.76) (Fig. 4c). Increasing the number of screws and filling all available screw holes in the plates did not increase the overall construct stiffness. The stiffness of the construct with all screws (3 through 13 filled) was 938 N/mm compared to 995 N/mm in the construct with only 3 screws (*p* = 0.24) (Fig. 4d).

Does fracture working length affect construct stiffness given the same plate material, length and type of screws? A larger space between the distal and proximal screw clusters did not change the overall construct stiffness. The stiffness of constructs with short working length of 5.4 cm (995 N/mm) was similar to those with long working length of 9.4 cm (944 N/mm, *p* = 0.24) (Fig. 4e).

Does plate material (titanium versus stainless steel) affect construct stiffness given the same fracture working length, plate length, type and number of screws? The use of stainless steel as opposed to titanium for plate material did not appear to influence overall stiffness. The stiffness of constructs with stainless steel plates (904 N/mm) was similar to those with titanium plates (943 N/mm, *p* = 0.12) (Fig. 4f).

## Discussion

Early reports on the success of LLP for supracondylar femur fractures have been tempered by more recent reports of high rates of nonunion [4–22]. In addition to patient and injury related factors such as obesity, diabetes, smoking and open injuries, fixation properties can contribute to the risk of nonunion [22, 29–34]. Construct stiffness can contribute to the biomechanical environment and influences fracture healing [30, 34, 35]. While the optimal stiffness to promote healing is unknown, it has been postulated that some configurations of lateral locked plates are too stiff and may cause difficulties with healing [21, 29, 36–38]. While there have been some technological advances, such as the concept of far cortical locking, to decrease construct stiffness, there is little data to help the surgeons influence construct stiffness when applying commonly available implants [29, 36, 38–40]. The purpose of this study is to evaluate the effect of surgeon-controlled factors on stiffness in employing LLP for supracondylar femur fractures fixation.

The primary limitation of this study is that the ideal stiffness for fracture fixation when applying lateral locked plates to the femur is unknown. Concerning this limitation, a 20 % difference in stiffness was chosen for power analysis as a pragmatic difference. While the results of this mechanical study provide guidance as to how to increase or decrease construct stiffness, it does not provide information about the ideal construct stiffness for bone healing. Furthermore, utilization of lateral locked plating, only in a specific type of distal femur fracture (OTA/AO type 33-A3) was evaluated, and other types of distal femur fractures and fixations were not assessed. In addition, the hybrid construct as a variation of utilizing locking and nonlocking screws was not evaluated. The model in this study primarily determines the stiffness of each osteosynthesis construct in its respective configuration under axial load; torsional and bending loads were not assessed in the current study. Another potential limitation is that results of this study are limited to the time zero of each construct, however as with any cadaveric or sawbone biomechanical study, there is an inherent time zero limitation, and these result does not investigate biologic factors such as biologic healing and muscle forces. In addition, cyclic loading was not implemented, which would have simulated the physiological scenario better. The synthetic bone itself most likely has very little effect on the construct stiffness, providing consistency between groups and allowing for better comparisons than would be possible with cadaveric specimens. However, as many of these injuries occur in the elderly, the magnitude of the effect of the changes on stiffness seen may be different in osteoporotic bones. Care should be taken in extrapolating the results to patients with poor bone quality, since some of the configurations in this study may result in early implant failure or cut out or cut through, in particular with bones with reduced quality. Finally, this study focused only on construct stiffness. Changes in plate length, screw type and number, and plate and screw material may also affect fatigue failure and load to catastrophic failure.

We found that longer plates created a stiffer construct when plate material, screw type and number, and fracture working length were kept constant. Ricci et al. have shown that short plate length is a risk factor for nonunion [24]. It may be that other factors accounted for the clinical difference seen – such as a lower load to failure or weakness in cyclical loading, or that stiffness is not the primary driver of nonunion in his series. For example, when shorter plates were used, the fracture working length was likely less, and that the longer plates had a high rate of union because they spanned a larger area of comminution, leading to decreased stiffness. Our data suggest that when all other variables are held constant, increasing plate length increases overall construct stiffness.

We found that the use of bicortical nonlocking screws is associated with a decrease in overall construct stiffness. This gives credence to the argument that nonlocking screws can be employed in the diaphysis, should quality of the bone stock allow it. This technique may be beneficial for allowing callus formation and indirect bone healing. There was no significant difference in stiffness comparing the use of bicortical and unicortical locking screws. This result is similar to the result of a study by Beingessner et al. that showed no effect on overall stiffness when comparing unicortical and bicortical locking screws in a similar construct [41]. However, the similarity in stiffness for unicortical and bicortical locking screws might change during cyclic or fatigue loading [35, 42]. Due to similar construct stiffness between unicortical and bicortical locking screws, unicortical locking plate may be a reasonable option in the treatment of distal femur fracture fixation to avoid complications associated with failure mode in periprosthetic femur fracture [43].

We didn’t find filling all of the holes of the plate to have an effect on axial stiffness. This was in accordance with previous research showing that all locked, all unlocked, proximal unlocked, and distally unlocked plating construct did not differ in axial stiffness [44]. This finding gives reassurance to surgeons who feel that they need to place additional screws in cases of osteoporotic bones, where additional fixation is required. As far as the most proximal screw hole is filled, the addition of extra screws in the diaphysis, well away from the fracture, does not appear to increase construct stiffness.

Surprisingly, increasing the fracture working length did not have an effect of stiffness. With a working length of 5.4 cm, the stiffness was 995 N/mm, while increasing the working length to 9.4 cm decreased the stiffness to only 938 N/mm, ( not statistically significant (*p* = 0.24)). In clinical practice, fractures have longer fracture line that can lead to a higher working length than tested for this study. In a retrospective review by W.H. Harvin et al., no significant difference in fracture union between shorter and higher working lengths was demonstrated. [45] Furthermore, this finding can be related to the loading conditions being purely axial and not identical to the physiological loading axis of the femur during walking. With oblique loading the different working lengths would have different moment arms and would thus presumably result in different construct stiffness [29, 36, 37]. However, it appears that the effect of the working length is less important than the type of screw used (bicortical locking vs. bicortical nonlocking) or the plate length. Our data suggest that it is the interface between the plate and the screws and bone that drives construct stiffness, with working length having a lesser effect.

Varying plate and screw material (titanium vs. stainless steel) did not appear to influence overall construct stiffness. This finding contradicts the findings of two previous studies that showed stainless steel had greater stiffness in comparison with titanium [35, 46]. However, they studied four constructs, all having locking screws, which may give them a higher power to discover small differences. In our study, the role of material properties on the stiffness may be dominated by other factors such as screw type and location, plate length, and plate geometry. The interface between the plate and the screws and bone appears to be more important than the flexibility of the plate in this fracture model. It may be that in fracture-fixation constructs with much larger working lengths, the lower modulus of elasticity of titanium has a lower overall stiffness. Distal femur fractures in the elderly often involve a significant amount of comminution and thus have a relatively large fracture working length. Further study is warranted to determine whether implant material effects stiffness in this scenario.

Many supracondylar fractures have large areas of comminution, requiring a large gap between the distal most diaphyseal screw and the distal screw cluster. In our previous experience with supracondylar femur fractures treated with LLP, titanium plates have had a lower risk of nonuion [22]. It may be that in cases with large fracture working lengths plate material becomes an important factor in overall fracture-fixation construct stiffness. Further, poor bone quality and osteoporosis likely influence stiffness as well. In our study we did not see a large effect of working length or plate material, but further study is required to better understand the effect of osteoporotic bone as well as cases of significantly larger fracture working lengths.

## Conclusions

Surgeons can control a number of variable to influence the overall construct stiffness during lateral plating of supracondular femur fractures. Using shorter plates and using bicortical nonlocking screws (vs. bicortical locking screws) reduces overall construct stiffness. However, using more screws, using unicortical locking screws, increasing fracture working length and varying plate material (titanium vs. stainless steel) does not appear to significantly alter construct stiffness. Our data suggest when additional screws are needed, they may be placed without increasing stiffness. However, this observation should be verified in cadaveric and clinical studies. While surgeons can adjust plate length and screw types to affect overall fracture-fixation construct stiffness the optimal stiffness to promote healing remains unknown.

## Data Availability

Data will be made available by request. Please contact the corresponding author Dr. Michael Weaver at mjweaver@bwh.harvard.edu.

## Abbreviations

LLP: Lateral locked plating
LISS: Less Invasive Stabilization System
AO/OTA: Arbeitsgemeinschaft für Osteosynthesefragen / Orthopaedic Trauma Association
ANOVA: Analysis of variance

## References

[1] Zlowodzki M, Bhandari M, Marek DJ, Cole PA, Kregor PJ. Operative treatment of acute distal femur fractures: systematic review of 2 comparative studies and 45 case series (1989 to 2005). J Orthop Trauma. 2006;20(5):366–71. doi:10.1097/00005131-200605000-00013. PubMed PMID: 16766943. Epub 2006/06/13. 10.1097/00005131-200605000-00013 16766943

[2] Meek RM, Norwood T, Smith R, Brenkel IJ, Howie CR. The risk of peri-prosthetic fracture after primary and revision total hip and knee replacement. J Bone Joint Surg Br. 2011;93(1):96–101. doi:10.1302/0301-620X.93B1.25087. PubMed PMID: 21196551. Epub 2011/01/05. 10.1302/0301-620X.93B1.25087 21196551

[3] Kubiak EN, Fulkerson E, Strauss E, Egol KA. The evolution of locked plates. J Bone Joint Surg Am. 2006;88(Suppl 4):189–200. doi:10.2106/JBJS.F.00703. PubMed PMID: 17142448. Epub 2006/12/05. 10.2106/JBJS.F.00703 17142448

[4] Kolb W, Guhlmann H, Windisch C, Marx F, Kolb K, Koller H. Fixation of distal femoral fractures with the Less Invasive Stabilization System: a minimally invasive treatment with locked fixed-angle screws. J Trauma. 2008;65(6):1425-34. Epub 2008/12/17. doi: 10.1097/TA.0b013e318166d24a. PubMed PMID: 19077637. 10.1097/TA.0b013e318166d24a 19077637

[5] Fulkerson E, Tejwani N, Stuchin S, Egol K. Management of periprosthetic femur fractures with a first generation locking plate. Injury. 2007;38(8):965–72. doi:10.1016/j.injury.2007.02.026. PubMed PMID: 17561020. Epub 2007/06/15. 10.1016/j.injury.2007.02.026 17561020

[6] Fankhauser F, Gruber G, Schippinger G, Boldin C, Hofer HP, Grechenig W, Szyszkowitz R (2004). Minimal-invasive treatment of distal femoral fractures with the LISS (Less Invasive Stabilization System): a prospective study of 30 fractures with a follow up of 20 months. Acta Orthop Scand.

[7] Haidukewych G, Sems SA, Huebner D, Horwitz D, Levy B. Results of polyaxial locked-plate fixation of periarticular fractures of the knee. J Bone Joint Surg Am. 2007;89(3):614–20. doi:10.2106/JBJS.F.00510. PubMed PMID: 17332111. Epub 2007/03/03. 10.2106/JBJS.F.00510 17332111

[8] Kao FC, Tu YK, Su JY, Hsu KY, Wu CH, Chou MC. Treatment of distal femoral fracture by minimally invasive percutaneous plate osteosynthesis: comparison between the dynamic condylar screw and the less invasive stabilization system. J Trauma. 2009;67(4):719–26. doi:10.1097/TA.0b013e31819d9cb2. PubMed PMID: 19820577. Epub 2009/10/13. 10.1097/TA.0b013e31819d9cb2 19820577

[9] Kregor PJ, Stannard J, Zlowodzki M, Cole PA, Alonso J. Distal femoral fracture fixation utilizing the Less Invasive Stabilization System (L.I.S.S.): the technique and early results. Injury. 2001;32 Suppl 3:SC32-47. Epub 2002/03/13. doi: 10.1016/s0020-1383(01)00182-6. PubMed PMID: 11888193. 10.1016/s0020-1383(01)00182-6 11888193

[10] Syed AA, Agarwal M, Giannoudis PV, Matthews SJ, Smith RM. Distal femoral fractures: long-term outcome following stabilisation with the LISS. Injury. 2004;35(6):599–607. Epub 2004/05/12. doi: 10.1016/S0020-1383(03)00247-X. PubMed PMID: 15135280. 10.1016/S0020-1383(03)00247-X 15135280

[11] Weight M, Collinge C. Early results of the less invasive stabilization system for mechanically unstable fractures of the distal femur (AO/OTA types A2, A3, C2, and C3). J Orthop Trauma. 2004;18(8):503–8. doi:10.1097/00005131-200409000-00005. PubMed PMID: 15475845. Epub 2004/10/12. 10.1097/00005131-200409000-00005 15475845

[12] Schutz M, Muller M, Kaab M, Haas N (2003). Less invasive stabilization system (LISS) in the treatment of distal femoral fractures. Acta Chir Orthop Traumatol Cech.

[13] Ricci AR, Yue JJ, Taffet R, Catalano JB, DeFalco RA, Wilkens KJ (2004). Less Invasive Stabilization System for treatment of distal femur fractures. Am J Orthop (Belle Mead NJ).

[14] Ricci WM, Loftus T, Cox C, Borrelli J. Locked plates combined with minimally invasive insertion technique for the treatment of periprosthetic supracondylar femur fractures above a total knee arthroplasty. J Orthop Trauma. 2006;20(3):190–6. doi:10.1097/00005131-200603000-00005. PubMed PMID: 16648700. Epub 2006/05/02. 10.1097/00005131-200603000-00005 16648700

[15] Wong MK, Leung F, Chow SP. Treatment of distal femoral fractures in the elderly using a less-invasive plating technique. Int Orthop. 2005;29(2):117–20. doi:10.1007/s00264-004-0609-3. PubMed PMID: 15703938; PMCID: PMC3474502. Epub 2005/02/11. 10.1007/s00264-004-0609-3 PMC3474502 15703938

[16] Kregor PJ, Stannard JA, Zlowodzki M, Cole PA. Treatment of distal femur fractures using the less invasive stabilization system: surgical experience and early clinical results in 103 fractures. J Orthop Trauma. 2004;18(8):509–20. doi:10.1097/00005131-200409000-00006. PubMed PMID: 15475846. Epub 2004/10/12. 10.1097/00005131-200409000-00006 15475846

[17] Schutz M, Muller M, Regazzoni P, Hontzsch D, Krettek C, Van der Werken C, Haas N. Use of the less invasive stabilization system (LISS) in patients with distal femoral (AO33) fractures: a prospective multicenter study. Arch Orthop Trauma Surg. 2005;125(2):102-8. Epub 2005/02/03. doi: 10.1007/s00402-004-0779-x. PubMed PMID: 15688230. 10.1007/s00402-004-0779-x 15688230

[18] Ebraheim NA, Liu J, Hashmi SZ, Sochacki KR, Moral MZ, Hirschfeld AG. High complication rate in locking plate fixation of lower periprosthetic distal femur fractures in patients with total knee arthroplasties. J Arthroplasty. 2012;27(5):809–13. doi: 10.1016/j.arth.2011.08.007. PubMed PMID: 21964235. 10.1016/j.arth.2011.08.007 21964235

[19] Henderson CE, Kuhl LL, Fitzpatrick DC, Marsh JL (2011).

[20] Streubel PN, Gardner MJ, Morshed S, Collinge CA, Gallagher B, Ricci WM. Are extreme distal periprosthetic supracondylar fractures of the femur too distal to fix using a lateral locked plate? J Bone Joint Surg Br. 2010;92(4):527–34. doi:10.1302/0301-620X.92B3.22996. PubMed PMID: 20357329. Epub 2010/04/02. 10.1302/0301-620X.92B3.22996 20357329

[21] Lujan TJ, Henderson CE, Madey SM, Fitzpatrick DC, Marsh JL, Bottlang M. Locked plating of distal femur fractures leads to inconsistent and asymmetric callus formation. J Orthop Trauma. 2010;24(3):156–62. doi:10.1097/BOT.0b013e3181be6720. PubMed PMID: 20182251. Epub 2010/02/26. 10.1097/BOT.0b013e3181be6720 20182251

[22] Rodriguez EK, Boulton C, Weaver MJ, Herder LM, Morgan JH, Chacko AT, Appleton PT, Zurakowski D, Vrahas MS. Predictive factors of distal femoral fracture nonunion after lateral locked plating: a retrospective multicenter case-control study of 283 fractures. Injury. 2014;45(3):554–9. doi:10.1016/j.injury.2013.10.042. PubMed PMID: 24275357. Epub 2013/11/28. 10.1016/j.injury.2013.10.042 24275357

[23] Henderson CE, Lujan TJ, Kuhl LL, Bottlang M, Fitzpatrick DC, Marsh JL. 2010 mid-America Orthopaedic Association Physician in Training Award: healing complications are common after locked plating for distal femur fractures. Clin Orthop Relat Res. 2011;469(6):1757–65. doi:10.1007/s11999-011-1870-6. PubMed PMID: 21424831; PMCID: PMC3094618. Epub 2011/03/23. 10.1007/s11999-011-1870-6 PMC3094618 21424831

[24] Ricci WM, Streubel PN, Morshed S, Collinge CA, Nork SE, Gardner MJ. Risk factors for failure of locked plate fixation of distal femur fractures: an analysis of 335 cases. J Orthop Trauma. 2014;28(2):83–9. doi:10.1097/BOT.0b013e31829e6dd0. PubMed PMID: 23760176. Epub 2013/06/14. 10.1097/BOT.0b013e31829e6dd0 23760176

[25] Chong AC, Friis EA, Ballard GP, Czuwala PJ, Cooke FW. Fatigue performance of composite analogue femur constructs under high activity loading. Ann Biomed Eng. 2007;35(7):1196 – 205. Epub 2007/03/29. doi: 10.1007/s10439-007-9284-z. PubMed PMID: 17390224. 10.1007/s10439-007-9284-z 17390224

[26] Chong ACM, Miller F, Buxton M, Friis EA (2007). Fracture Toughness and Fatigue Crack Propagation Rate of Short Fiber Reinforced Epoxy Composites for Analogue Cortical Bone. J Biomech Eng.

[27] Heiner AD. Structural properties of fourth-generation composite femurs and tibias. J Biomech. 2008;41(15):3282–4. doi:10.1016/j.jbiomech.2008.08.013. PubMed PMID: 18829031. Epub 2008/10/03. 10.1016/j.jbiomech.2008.08.013 18829031

[28] Marsh JL, Slongo TF, Agel J, Broderick JS, Creevey W, DeCoster TA, Prokuski L, Sirkin MS, Ziran B, Henley B, Audige L. Fracture and dislocation classification compendium – 2007: Orthopaedic Trauma Association classification, database and outcomes committee. J Orthop Trauma. 2007;21(10 Suppl):1–133. doi:10.1097/00005131-200711101-00001. PubMed PMID: 18277234. Epub 2008/03/07. 10.1097/00005131-200711101-00001 18277234

[29] Bottlang M, Schemitsch CE, Nauth A, Routt M Jr, Egol KA, Cook GE, Schemitsch EH. Biomechanical Concepts for Fracture Fixation. J Orthop Trauma. 2015;29(Suppl 12):28–33. doi:10.1097/BOT.0000000000000467. PubMed PMID: 26584263; PMCID: PMC4654707. Epub 2015/11/20. 10.1097/BOT.0000000000000467 PMC4654707 26584263

[30] Panteli M, Rodham P, Giannoudis PV. Biomechanical rationale for implant choices in femoral neck fracture fixation in the non-elderly. Injury. 2015;46(3):445–52. doi:10.1016/j.injury.2014.12.031. PubMed PMID: 25597514. Epub 2015/01/20. 10.1016/j.injury.2014.12.031 25597514

[31] Gurusamy K, Parker MJ, Rowlands TK. The complications of displaced intracapsular fractures of the hip: the effect of screw positioning and angulation on fracture healing. J Bone Joint Surg Br. 2005;87(5):632–4. doi:10.1302/0301-620X.87B5.15237. PubMed PMID: 15855363. Epub 2005/04/28. 10.1302/0301-620X.87B5.15237 15855363

[32] Riehl JT, Koval KJ, Langford JR, Munro MW, Kupiszewski SJ, Haidukewych GJ. Intramedullary nailing of subtrochanteric fractures–does malreduction matter? Bull Hosp Jt Dis (2013). 2014;72(2):159 – 63. Epub 2014/08/26. PubMed PMID: 25150344. 25150344

[33] Clement ND, Goudie EB, Brooksbank AJ, Chesser TJ, Robinson CM. Smoking status and the Disabilities of the Arm Shoulder and Hand score are early predictors of symptomatic nonunion of displaced midshaft fractures of the clavicle. Bone Joint J. 2016;98-B(1):125 – 30. Epub 2016/01/07. doi: 10.1302/0301-620X.98B1.36260. PubMed PMID: 26733525. 10.1302/0301-620X.98B1.36260 26733525

[34] Kakar SET, Browner BD (2008). The Biology and Enhancement of Skeletal Repair. Skeletal Trauma: Basic Science, Management and Reconstruction.

[35] Schmidt U, Penzkofer R, Bachmaier S, Augat P. Implant material and design alter construct stiffness in distal femur locking plate fixation: a pilot study. Clin Orthop Relat Res. 2013;471(9):2808–14. doi:10.1007/s11999-013-2867-0. PubMed PMID: 23436162; PMCID: PMC3734410. Epub 2013/02/26. 10.1007/s11999-013-2867-0 PMC3734410 23436162

[36] Mardian S, Schmolz W, Schaser KD, Duda GN, Heyland M. Interfragmentary lag screw fixation in locking plate constructs increases stiffness in simple fracture patterns. Clin Biomech (Bristol Avon). 2015;30(8):814–9. doi: 10.1016/j.clinbiomech.2015.06.008. PubMed PMID: 26094776. 10.1016/j.clinbiomech.2015.06.008 26094776

[37] Mardian S, Schaser KD, Duda GN, Heyland M. Working length of locking plates determines interfragmentary movement in distal femur fractures under physiological loading. Clin Biomech (Bristol Avon). 2015;30(4):391–6. doi:10.1016/j.clinbiomech.2015.02.006. PubMed PMID: 25716162. Epub 2015/02/27. 10.1016/j.clinbiomech.2015.02.006 25716162

[38] MacLeod AR, Simpson AH, Pankaj P. Reasons why dynamic compression plates are inferior to locking plates in osteoporotic bone: a finite element explanation. Comput Methods Biomech Biomed Engin. 2015;18(16):1818–25. doi: 10.1080/10255842.2014.974580. PubMed PMID: 25473732. 10.1080/10255842.2014.974580 25473732

[39] Moazen M, Leonidou A, Pagkalos J, Marghoub A, Fagan MJ, Tsiridis E. Application of Far Cortical Locking Technology in Periprosthetic Femoral Fracture Fixation: A Biomechanical Study. J Arthroplasty. 2016;31(8):1849–56. doi:10.1016/j.arth.2016.02.013. PubMed PMID: 26989031. Epub 2016/03/19. 10.1016/j.arth.2016.02.013 26989031

[40] Katthagen JC, Schwarze M, Warnhoff M, Voigt C, Hurschler C, Lill H. Influence of plate material and screw design on stiffness and ultimate load of locked plating in osteoporotic proximal humeral fractures. Injury. 2016;47(3):617–24. doi:10.1016/j.injury.2016.01.004. PubMed PMID: 26804939. Epub 2016/01/26. 10.1016/j.injury.2016.01.004 26804939

[41] Beingessner D, Moon E, Barei D, Morshed S. Biomechanical analysis of the less invasive stabilization system for mechanically unstable fractures of the distal femur: comparison of titanium versus stainless steel and bicortical versus unicortical fixation. J Trauma. 2011;71(3):620–4. doi:10.1097/TA.0b013e31820337c4. PubMed PMID: 21610539. Epub 2011/05/26. 10.1097/TA.0b013e31820337c4 21610539

[42] Beltran MJ, Gary JL, Collinge CA (2015). Management of distal femur fractures with modern plates and nails: state of the art. J Orthop Trauma.

[43] Gwinner C, Mardian S, Droge T, Schulze M, Raschke MJ, Stange R. Bicortical screw fixation provides superior biomechanical stability but devastating failure modes in periprosthetic femur fracture care using locking plates. Int Orthop. 2015;39(9):1749–55. doi:10.1007/s00264-015-2787-6. PubMed PMID: 25947899. Epub 2015/05/08. 10.1007/s00264-015-2787-6 25947899

[44] Cui S, Bledsoe JG, Israel H, Watson JT, Cannada LK. Locked plating of comminuted distal femur fractures: does unlocked screw placement affect stability and failure? J Orthop Trauma. 2014;28(2):90–6. doi:10.1097/BOT.0b013e31829f9504. PubMed PMID: 23860132. Epub 2013/07/19. 10.1097/BOT.0b013e31829f9504 23860132

[45] Harvin WH, Oladeji LO, Della Rocca GJ, Murtha YM, Volgas DA, Stannard JP, Crist BD. Working length and proximal screw constructs in plate osteosynthesis of distal femur fractures. Injury. 2017;48(11):2597–601. doi: 10.1016/j.injury.2017.08.064. PubMed PMID: 28889934. 10.1016/j.injury.2017.08.064 28889934

[46] Kandemir U, Augat P, Konowalczyk S, Wipf F, von Oldenburg G, Schmidt U. Implant Material, Type of Fixation at the Shaft, and Position of Plate Modify Biomechanics of Distal Femur Plate Osteosynthesis. J Orthop Trauma. 2017;31(8):e241-e6. doi:/BOT.0000000000000860. PubMed PMID: 28394844. Epub 2017/04/11. 10.1097/BOT.0000000000000860 28394844

